# Biological profile of medical response in alcoholic patients of different ethnic groups in Siberia

**DOI:** 10.1192/j.eurpsy.2021.1814

**Published:** 2021-08-13

**Authors:** T. Shushpanova, T. Novozheeva, N. Bokhan, M. Belousov, E. Bezverkhnyaya, N. Kolomiets, A. Mandel

**Affiliations:** 1 Clinical Psychoneuroimmunology And Neurobiology Lab, Mental Health Research Institute, Tomsk, Russian Federation; 2 Neurobiology, Mental Health Research Institute Tomsk National Research Medical Center Russia Academy of Science, Tomsk, Russian Federation; 3 The Department Of Addictive States, Mental Health Research Institute, Tomsk National Research Medical Center of the Russian Academy of Sciences, Tomsk, Russian Federation; 4 Department Of Pharmaceutical Analysis, Siberian State Medical University, Tomsk, Russian Federation; 5 Institute Of Physics Of High Technologies, Tomsk Polytechnic University, Tomsk, Russian Federation

## Abstract

**Introduction:**

The individual sensitivity of a person to the effects of alcohol is defined as the possibility of adaptive reactions, which are controlled by various factors associated in their manifestation with characteristics in various ethnic populations.

**Objectives:**

To determine biological profile of medical response in alcoholic patients of different ethnic groups.

**Methods:**

168 alcoholic men, aged 17 to 62 years were examined. For the therapeutic correction of withdrawal and post-withdrawal symptoms of patients from two different ethnic groups (Tatars and Russians in Siberia), the original anticonvulsant galodif (M-chloro-benzhydrylurea) was used. Pharmacokinetic parameters were calculated a model-independent method of statistical moments: half-life (T1/2,h), total clearance (Clt,ml/min), average time of the residual drug in the body (MRI,h), average elimination time (MET,h), the area under the pharmacokinetic curve (AUC, μg/ml).

**Results:**

Galodif causes a reduction in almost half T1/2, a significant decrease in the average time of the residual drug in the body MRI, and the average elimination time MET. Overall clearance increased. Under the influence of the course with Galodif, antipyrine elimination intensified, which indicates the induction of the cytochrome P-450 system of microsomal liver monooxygenases in Russian alcoholic patients. Galodif causes a reduction of almost five-fold T1/2, a significant decrease MRI and MET. Activation of oxidative metabolism of xenobiotics in Tatar alcoholic patients is more significant.

**Conclusions:**

The increased sensitivity of P-450 cytochrome system to anticonvulsants action with inductive detoxification properties reflects differences in adaptive mechanisms in human from various ethnic groups, what is significant in the therapy.

**Disclosure:**

No significant relationships.

